# Curaxin CBL0137 eradicates drug resistant cancer stem cells and potentiates efficacy of gemcitabine in preclinical models of pancreatic cancer

**DOI:** 10.18632/oncotarget.2701

**Published:** 2014-11-06

**Authors:** Catherine Burkhart, Daria Fleyshman, Rachael Kohrn, Mairead Commane, Jennifer Garrigan, Vadim Kurbatov, Ilya Toshkov, Rajesh Ramachandran, Laura Martello, Katerina V. Gurova

**Affiliations:** ^1^ Buffalo Biolabs, LLC, Buffalo, NY, USA; ^2^ Department of Cell Stress Biology, Roswell Park Cancer Institute, Buffalo, NY, USA; ^3^ Division of Gastroenterology & Hepatology, SUNY Downstate Medical Center, Brooklyn, NY, USA

**Keywords:** curaxins, CBL0137, pancreatic cancer, gemcitabine, SSRP1, SPT16, cancer stem cells

## Abstract

Pancreatic ductal adenocarcinoma (PDA) continues to be one of the deadliest cancers due to the absence of effective treatment. Curaxins are a class of small molecules with anti-cancer activity demonstrated in different models of cancer in mice. The lead curaxin compound, CBL0137, recently entered Phase I clinical trials. Curaxins modulate several important signaling pathways involved in the pathogenesis of PDA through inhibition of chromatin remodeling complex FACT. FACT is overexpressed in multiple types of tumor, with one of the highest rate of overexpression in PDA (59%). In this study, the efficacy of CBL0137 alone or in combination with current standard of care, gemcitabine, was tested against different models of PDA *in vitro* and in mouse models. It was found that CBL0137 alone is a potent inducer of apoptosis in pancreatic cancer cell lines and is toxic not only for proliferating bulk tumor cells, but also for pancreatic cancer stem cells. In mice, CBL0137 was effective against several PDA models, including orthotopic gemcitabine resistant PANC-1 model and patient derived xenografts, in which CBL0137 anti-tumor effect correlated with overexpression of FACT. Moreover, we observed synergy of CBL0137 with gemcitabine which may be explained by the ability of CBL0137 to inhibit several transcriptional programs induced by gemcitabine, including NF-kappaB response and expression of ribonucleotide reductase, one of the targets of gemcitabine in cells. This data suggest testing of CBL0137 efficacy in Phase II trial in PDA patients alone and in combination with gemcitabine.

## INTRODUCTION

Mortality from pancreatic cancer is close to 100% due to the absence of effective treatment approaches. Current frontline therapies of gemcitabine + nab-paclitaxel, or FOLFINOX (infusional 5-fluorouracil, leucovorin, irinotecan and oxaloplatin) have demonstrated some progress in treatment above gemcitabine, the standard of care agent (median overall survival 8.5-11 months vs ~6 months for gemcitabine), however, patients will ultimately present with progressive disease and many patients will not be eligible for FOLFINOX due to its toxicity [1-3]. Thus, novel therapies are urgently needed.

CBL0137 is a member of a new class of recently discovered candidate anti-cancer agents, named curaxins, that modulate several important signaling pathways involved in the pathogenesis of pancreatic ductal adenocarcinoma (PDA, [4]). In particular, CBL0137 and related molecules can simultaneously activate p53 and inhibit cellular stress pathways mediated by NF-κB and HSF-1 [4], [5, 6]. One of the most significant factors predisposing patients to PDA is chronic pancreatic inflammation accompanied by constitutive activity of NF-κB (reviewed in [7]). In addition, the heat shock response stress pathway, which is mediated by HSF1, is also frequently overactive in PDA cells [8]. The effects of CBL0137 on these pathways, culminating in tumor cell death, are mediated by the inhibition of FACT [4], a chromatin remodeling complex composed of SSRP1 and SPT16 subunits, that is involved in the transcription of genes with highly ordered chromatin structure, replication, and mitosis [9-11]. Curaxins are indirect inhibitors of FACT: they bind DNA without causing DNA breaks or any other sort of damage and without activating DNA damage sensitive signaling pathways [4]. They do however cause topological alteration in the DNA helix to which FACT is sensitive. FACT binds with high affinity to DNA in the presence of curaxins and is unable to bind histones to perform its normal chromatin remodeling function [4].

FACT is expressed during early embryogenesis and in undifferentiated progenitors and stem cells of adult tissues while protein levels of both FACT subunits are almost undetectable in differentiated cells of adult tissues [12]. FACT is overexpressed in several tumor types compared to equivalent normal tissues. In particular, SSRP1 is expressed in a high proportion of lung and pancreatic cancers (~45-59%) [13]. FACT positive tumors are associated with an aggressive malignant phenotype (high grade, metastatic disease, worse overall survival) [13]. Therefore, FACT represents a potentially important target for cancer therapy. Taken together, these data suggested that CBL0137 may be effective against PDA.

In the studies presented here, the levels of the indirect CBL0137 target, FACT (SSRP1 and SPT16 subunits), were examined in patient PDA surgical samples and the effect of CBL0137 monotherapy or combination with gemcitabine was evaluated using patient derived PDA xenografts and PANC-1 orthotopic tumors. In addition, potential mechanisms for the combined efficacy observed between CBL0137 and gemcitabine were investigated. CBL0137 was efficacious against mouse models of PDA and enhanced the effect of gemcitabine by causing a significant delay in tumor regrowth following the completion of treatment. The data presented here suggests that the combined effects may be a result of CBL0137 targeting of PDA stem cells as well as its modulation of the expression of genes that affect gemcitabine sensitivity in PDA cells. Together, these data indicate that CBL0137 may provide a clinical benefit for the treatment of PDA, particularly when combined with gemcitabine.

## RESULTS

### CBL0137 is toxic for gemcitabine-sensitive and resistant pancreatic ductal adenocarcinoma cells

**Figure 1:**
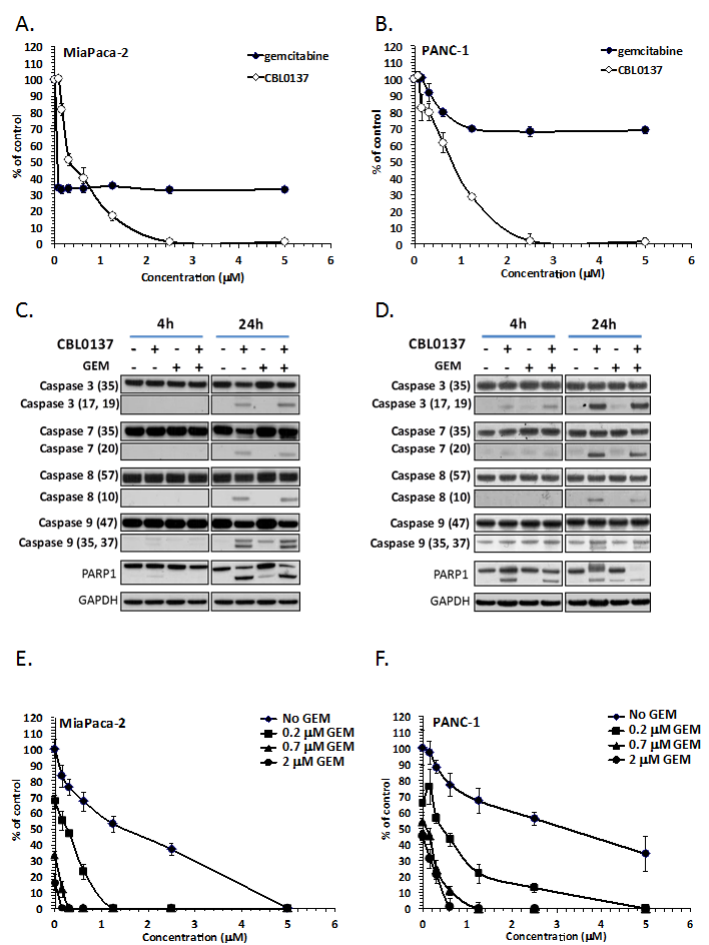
CBL0137 and gemcitabine toxicity to pancreatic ductal adenocarcinoma cell lines A-B. Viability of MiaPaCa-2 (A) and PANC-1 (B) cells incubated with different concentrations of CBL0137 for 24 hrs or gemcitabine for 72 hrs assessed 72 hours after start of treatment using Cell Titer Blue assay (Promega). Mean of three replicates +/− SDV. C-D. Detection of caspases 3, 7, 8, 9 and PARP1 cleavage in MiaPaCa-2 (C) or BxPC-3 (D) treated for 4 or 24 hrs with 2μM of CBL0137, 20μM of gemcitabine or their combination using immunoblotting with indicated antibodies (in parenthesis size of detected band in kDa). E-F. Colony forming assay using MiaPaCa-2 (E) or PANC-1 (F) cells treated for 4 hrs with different concentrations of CBL0137, gemcitabine or their combination. Mean of three replicates +/− SDV.

To test the effect of CBL0137 on gemcitabine-sensitive and -resistant PDA cells, MiaPaCa-2 and PANC-1 human PDA cell lines were used, which are gemcitabine-sensitive and resistant, respectively [14]. Both cell lines were sensitive to CBL0137 in 72h viability assays (Fig.1A, B). Importantly, while treatment with CBL0137 led to complete absence of living cells at concentrations above 2.5 μM, gemcitabine treatment, which as reported was more effective against MiaPaCa-2 than PANC-1 cells, resulted in growth arrest rather than cell death since no reduction in the number of living cells was observed with dose escalation (Fig.1A, B). Consistent with this observation, we did not see any biochemical signs of cell death, such as caspase activation or PARP1 cleavage, when we analyzed extracts of cells treated with gemcitabine using immunoblotting, while the same signs were evident in extracts of cells treated with CBL0137 (Fig.1C, D).

To test if CBL0137 could increase the toxicity of gemcitabine to sensitive and resistant cells, a colony forming assay was performed in which MiaPaCa-2 and PANC-1 cells which were treated for 4h with either drug alone or their combination. Surprisingly, CBL0137 caused a greater reduction in the number of colonies formed of not only MiaPaCa-2 cells when combined with gemcitabine, but also gemcitabine-resistant PANC-1 cells (Fig.1E, F). Thus, CBL0137 is toxic for pancreatic cancer cells independently of their sensitivity to gemcitabine and, moreover, is able to increase the sensitivity of both gemcitabine-sensitive and resistant PDA cells to gemcitabine.

### Anti-tumor effect of CBL0137 on gemcitabine-resistant tumor in mice

**Figure 2:**
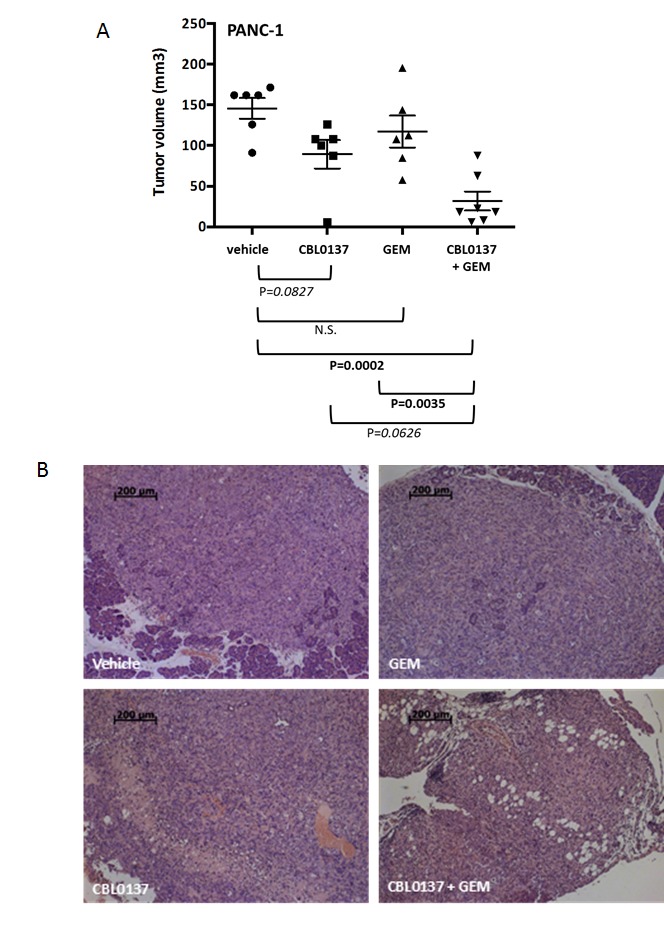
Effect of CBL0137 and gemcitabine on orthotopic PANC1 pancreatic tumor growth in nude mice PANC-1 cells were inoculated into the pancreas tail of nude mice (n=6-7/group). Two weeks following inoculation, treatment began with vehicle, 90 mg/kg CBL0137 i.v. 1/week, 40 mg/kg gemcitabine (GEM) i.p. Q4d or combination of CBL0137 and gemcitabine. Mice were treated for 4 weeks. One week following treatment, mice were euthanized and tumors measured and collected. (A) Scatter plot of tumor volumes for orthotopic PANC-1 tumors. Black bar represents the mean tumor volume for each treatment group. Error bars represent the standard error of the means. Comparisons across groups were performed using ANOVA. P values indicated in bold are statistically significant (P<0.05). Those in italics are approaching significance. (B) Histological assessment of the pancreas of PANC-1 tumor bearing athymic nude mice. Multiple serial sections from each mouse pancreas were analyzed for the presence of tumor. Representative H&E stained images for each treatment group are presented.

In order to determine whether the effect of CBL0137 monotherapy and combination with gemcitabine occurred *in vivo,* an orthotopic model of PANC-1, in which PANC-1 cells were inoculated directly into the tail of the pancreas of athymic nude mice, was utilized. Two weeks after inoculation, mice were treated for 4 weeks with 90 mg/kg CBL0137 intravenously (i.v.) once per week, 40 mg/kg gemcitabine intraperitoneally (i.p.) every 4^th^ day (Q4d) or a combination of the two agents. A fourth treatment group received only the corresponding vehicles. One week following the end of treatment, mice were euthanized and tumors of the pancreas measured and then collected for histological analysis. While CBL0137 and gemcitabine monotherapy had only a modest effect on PANC-1 orthotopic tumor growth, which failed to reach statistical significance (39% and 20% growth inhibition, respectively), the combination of the two agents caused a substantial decrease in PANC-1 tumor growth (78% growth inhibition, P=0.0002; Fig. 2A). Histological examination of multiple sections of the pancreatic tissues from each mouse confirmed the anti-tumor effect of CBL0137 monotherapy and the combination and a more minor effect by gemcitabine (Fig. 2B). Based on the analysis, the vehicle control tumors were actively growing with numerous mitoses present. There were almost no apoptotic bodies and no evidence of necrosis or infiltration of lymphoid cells (Fig. 2B). There was also extensive tumor growth observed in the pancreases of the gemcitabine monotherapy mice with only single apoptotic tumor cells visible (Fig. 2B). In contrast, the CBL0137 monotherapy group and the CBL0137-gemcitabine combination group samples showed large necrotic fields, numerous apoptotic bodies and loss of tumor cells. In addition, there was infiltration of lymphoid cells into and adjacent to the remaining tumor (Fig. 2B). Thus CBL0137 demonstrated an anti-tumor effect in gemcitabine-resistant tumors and also potentiated the anti-tumor efficacy of gemcitabine when used in combination.

### Anti-tumor activity of CBL0137 against patient derived xenografts of pancreatic ductal adenocarcinoma in mice

In addition to testing the *in vivo* efficacy of CBL0137 against a gemcitabine resistant orthotopic model, its efficacy was tested against more clinically relevant models of PDA, namely patient derived PDA tumors grown in SCID mice. Since patient samples represent closely the natural heterogeneity and variability of PDA in the clinic, the use of patient derived xenograft (PDX) models not only allowed for the evaluation of the anti-tumor efficacy of CBL0137 in general, but also whether or not the expression of the indirect target of CBL0137, FACT, correlates with tumor response to CBL0137.

**Figure 3:**
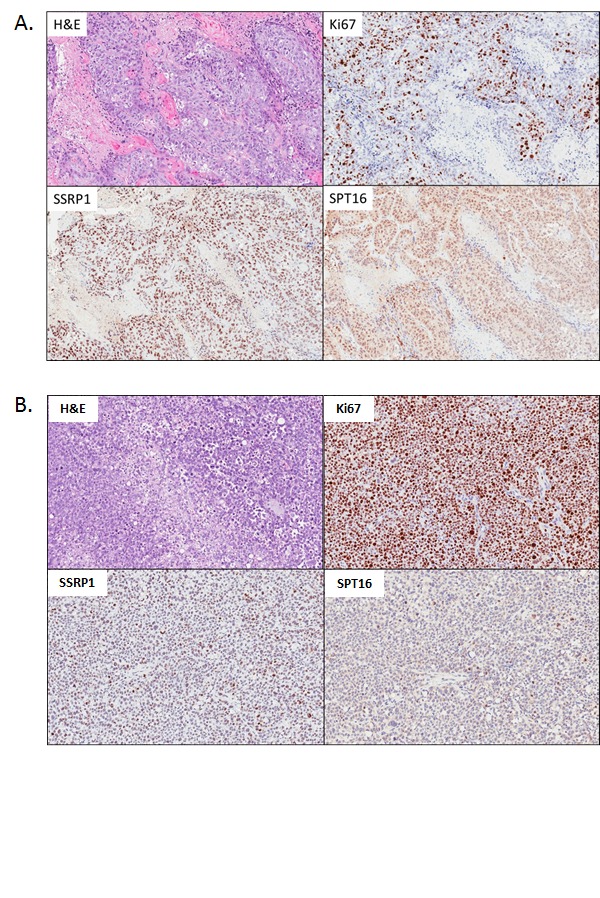
Morphology and expression of FACT subunits (SSRP1, SPT16) and proliferation marker Ki67 in PDX samples of pancreatic ductal adenocarcinoma (PDA) used in the study H&E and IHC staining with indicated antibodies. A. PDA#10978 with the highest score of FACT expression. B. PDA#13756 with the lowest score of FACT expression.

It was previously shown that the toxicity of CBL0137 to syngeneic pairs of tumor cells *in vitro* was dependent on the level of FACT [4]. However, the level of FACT in these cells was artificially reduced or increased, which may not reflect the natural dependence of cells on FACT. Moreover, inhibition of FACT expression *in vitro* is toxic for tumor cells and, therefore, no FACT-null cells could be generated for testing whether CBL0137 has anti-tumor effect in the absence of FACT. Furthermore, a naturally occurring FACT-negative cell line was not found among multiple cell lines of different origin that were tested *in vitro* (unpublished data). At the same time, FACT was present in only 59% of PDA samples from patients as judged by the immunohistochemical (IHC) staining of tissue microarrays (TMA) for the FACT subunit, SSRP1 [13]. Therefore, the dependence of CBL0137 anti-tumor activity on FACT subunit expression was tested *in vivo* against a panel of patient-derived pancreatic cancer xenografts (PDX) with varied expression of FACT subunits (Table 1). Upon receipt of a sample, part of the tumor was taken for IHC analysis of SSRP1 and SPT16 subunits and the remaining tumor was implanted into donor SCID mice. In addition, tumor was collected after passage in donor mice before transplantation into recipient mice. Based on TMA staining consisting of biopsy samples [13], it was expected that approximately half of the obtained patient samples would be negative for FACT. Surprisingly, however, all 10 samples were positive for SSRP1 and SPT16 (Table 1) and the levels of expression did not significantly change after passaging of tumors in SCID mice (Fig.S1). Staining was highly specific since each slide contained a mixture of positive (tumor cells) and negative (some tumor and stroma cells) cells (Fig. 3A, B and S1). Although there was a high similarity between the patterns of SSRP1 and SPT16 staining in the samples, there was no similarity between the staining of FACT subunits and Ki67 staining (Fig. 3 and S1). Furthermore, a comparison of the scores for intensity of staining and proportion of SSRP1/SPT16 or Ki67 positive cells revealed a significant positive correlation (r>0.6 and p<0.05) only between SSRP1 and SPT16 staining, but not between any of them and Ki67 staining (Table 1), confirming our previous observation made using normal tissues that FACT is not a marker of proliferating cells [12]. Interestingly, although not highly significant, some correlations were observed between time to engraftment (start of growth) of PDX in donor mice and Ki67 or SSRP1/SPT16 scores. This correlation was negative for Ki67 as expected, suggesting that samples with high proliferation rate start growing faster, but positive for SSRP1/SPT16, i.e. highly FACT positive samples, engrafted slower (Table 1). This again indicated that FACT is not a marker of quickly proliferating cells.

**Table 1:** Characteristics of PDX samples used in the study

PDX#	Growth^*^ (days to engraftment)	SSRP1 staining (scores^**^)			SPT16 staining (scores)			Ki67
		frequency	intensity	score	frequency	intensity	score	% positive cells
12274	74	3	3	9	3	2	6	45
15010	42	2	2	4	2	1	2	25
13047	no	3	3	9	3	3	9	NA
13756	28	1	2	2	1	2	2	95
12461	86	3	3	9	3	3	9	50
12298	no	NA	NA	NA	NA	NA	NA	NA
12914	86	3	3	9	2	2	4	30
13590	42	3	2	6	2	2	4	70
10978	113	3	3	9	3	3	9	60
12989	no	3	3	9	2	2	4	NA
r (growth)		*0.36*	*0.49*	*0.46*	0.23	*0.32*	*0.31*	*-0.36*
r (SSRP1)					**0.75**	**0.63**	**0.73**	*-0.47*
r (SPT16)								−0.07
*p* (growth)		0.31	0.15	0.18	0.52	0.37	0.39	0.31
*p* (SSRP1)					**0.01**	**0.05**	**0.017**	**0.17**
*p* (SPT16)								0.85

* time from surgery to the start of growth of any first subcutaneous tumor in donor mice detected by measurement of tumor volume.

** scoring was done as previously described [13].

r Pearson correlation coefficient between parameter indicated in the parenthesis and parameter in the column above. Absolute values of r>0.5 indicate strong correlation and are shown in bold, absolute values of r>0.3, but less than 0.5 indicate moderate correlation and are shown in italics.

p p-value of significance of correlation.

Based on this data, the efficacy of CBL0137 as a single agent was determined using four independent PDX samples with varying levels of FACT expression (Table 1): PDX #10978 (high FACT expression, SSRP1/SPT16 scores 9/9, Fig.3A), PDX #13590 (intermediate FACT expression, SSRP1/SPT16 scores 6/4, Fig.S1A), PDX #15010 (lower FACT expression, SSRP1/SPT16 scores 4/2, Fig.S1A), and PDX #13756 (the lowest FACT expression, SSRP1/SPT16 scores 2/2, Fig.3B). Three of the four PDX samples responded to CBL0137 treatment (Table 2). The response varied from partial regression (#10978, very high FACT) to suppression of tumor growth (#13590 and #15010 intermediate and low levels of FACT, respectively). In contrast, PDX #13756 with the lowest level of FACT among the four samples had little or no response to CBL0137 treatment (26% growth inhibition). Thus, there was a tendency for a higher response to CBL0137 monotherapy from PDX tumors with higher levels of FACT compared to tumor with the lowest level of FACT. This trend necessitates a study of the correlation between FACT expression in PDA tumors and patient response to CBL0137 in clinical trials.

**Table 2:** CBL0137 anti-tumor activity and expression of FACT in PDA tumors in mice

PDA#	Growth of tumors in control group (folds*)	Growth of tumors in CBL0137 treated group (folds)	Suppression of tumor growth by CBL0137**	Level of FACT
13756	39+/−11	29+/−12	26%	Low
13590	4.8+/−1.7	2.5+/−1	60%	Intermediate low
15010	25.5+/−11	15.5+/−10.5	40%	Intermediate
10978	1.89+/−0.85	0.87+/−0.42	115%	High

* is calculated by dividing tumor volume at 1 week after the end of treatment to tumor volume at day 1 of treatment.

** is calculated using formula: 100% × (ΔVolcontrol - ΔVoltreated)/ΔVolcontrol, where ΔVol = final tumor volume - initial tumor volume.

### Effect of CBL0137 on the sensitivity of pancreatic PDX models to gemcitabine

In order to better understand the potential clinical benefits of CBL0137, the efficacy of CBL0137 combined with gemcitabine, was evaluated using PDX #13756 and #13590 models described above. For these studies, tumor bearing mice received the following treatments for up to 4 weeks: 1) vehicle, 2) 40 mg/kg gemcitabine i.p. Q4d, 3) 80-90 mg/kg CBL0137 i.v. once per week, 4) gemcitabine + CBL0137. The combination was administered at the same doses and schedules as indicated for the monotherapies.

**Figure 4:**
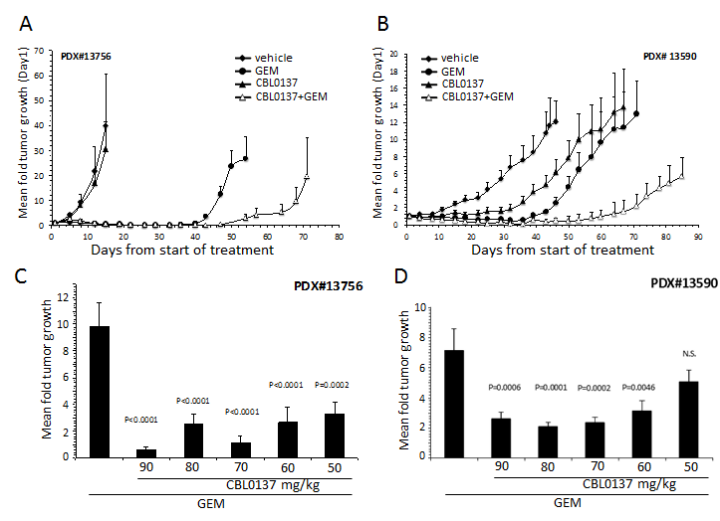
Effect of CBL0137 and gemcitabine on patient derived PDA xenograft models PDX#13756 or #13590 were inoculated into each flank of SCID mice (n=5/group (A,B) or n=10/group (C,D)). When at least one tumor per mouse reached ~50 mm3, treatment began with vehicle, 50-90 mg/kg CBL0137 i.v. 1/week, 20 mg/kg gemcitabine (GEM) i.p. Q4d or combination of CBL0137 and gemcitabine. Mice were treated for 4 weeks. Mice were followed for up to 90 days from start of treatment or when at least one tumor per mouse reached 1000 mm3. (A, B) Mean fold tumor growth was calculated by normalizing the tumor volume on Day X to that on Day 1 for each individual tumor and then averaging the normalized values for all tumors in each group at the designated time points. Error bars represent the standard error of the means. (C, D) Comparison of effect of gemcitabine or varying doses of CBL0137 in combination of gemcitabine on tumor growth three weeks (C) or four weeks (D) after treatment ended. Error bars represent the standard error of the means. Comparison across groups were made using ANOVA (P<0.05 is significant; N.S. not significant).

PDX #13756 was particularly sensitive to gemcitabine with 75-100% of tumors in all groups receiving gemcitabine as part of their treatment regimens either regressing or disappearing by Day 15 of treatment (Fig. 4A). Consistent with initial testing of CBL0137 against PDX#13756, CBL0137 monotherapy had little effect on tumor growth in this experiment (Fig. 4A). Surprisingly, CBL0137, although inactive on its own, enhanced the effect of gemcitabine on tumors as demonstrated by an increased latency for tumor regrowth (Fig. 4A). This effect was further illustrated by increasing the median survival time (MST) from 54 days for the gemcitabine only group to 78 days for its combination with CBL0137 (P=0.0153, LogRank test).

In contrast to PDX #13756, PDX #13590 was sensitive to both monotherapies with CBL0137 only slightly less efficacious than gemcitabine (Fig. 4B). The majority of tumors from mice of gemcitabine treatment group demonstrated tumor regression or complete disappearance by the end of the treatment period. Such regression was only observed with 30% of tumors in mice of the CBL0137 monotherapy group. Similarly to the #13756 study, the addition of CBL0137 to the gemcitabine regimen increased the latency of tumor regrowth compared to gemcitabine alone such that tumors implanted in four of the five mice in the CBL0137-gemcitabine combination group failed to reach the tumor size endpoint of 1000 mm^3^ two months after the end of treatment (mean tumor volume Day 88 222.7 ± 67.2 mm^3^). In contrast, 3 of the 4 mice in the gemcitabine monotherapy group were euthanized between days 73-79 from start of treatment due to at least one tumor of a mouse reaching the tumor size endpoint.

### CBL0137 enhances gemcitabine activity at sub-optimal doses

The studies described above were performed at or near the maximum tolerated dose (MTD) for once per week i.v. administration of CBL0137. In order to understand whether the effect of CBL0137 on the sensitivity of PDA to gemcitabine can be mediated at doses below the MTD, which would be beneficial in clinical trials, dose titration studies were performed for CBL0137 alone or in combination with gemcitabine using the PDX #13756 and #13590 models. Tumor bearing mice received 50-90 mg/kg CBL0137 i.v. once per week in the presence or absence of 20 mg/kg gemcitabine administered i.p. Q4d for up to 4 weeks and generated data was compared to the vehicle only treatment group. Sub-optimal doses of 50-60 mg/kg CBL0137 caused similar enhancement of gemcitabine antitumor activity as that produced by the MTD dose of 90 mg/kg as indicated by the lack of statistically significant differences among the combination groups (Fig. 4C, D). A similar enhancement of gemcitabine antitumor activity was achieved with orally delivered CBL0137 in the PDX #13756 model (Fig. S2A). Furthermore, similar results were achieved with the CBL0137-gemcitabine combination using H1975 non-small cell lung cancer xenografts, indicating that the combination effect is not specific to PDA (Fig. S2B).

### CBL0137 influences different mechanisms of pancreatic cancer resistance to gemcitabine

**Figure 5:**
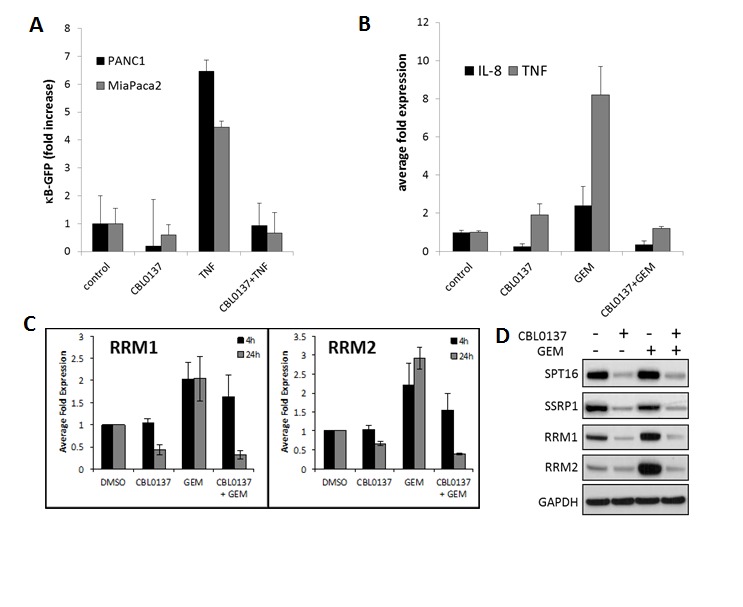
CBL0137 inhibit gemcitabine induced transcriptional responses A. CBL0137 inhibits activity of NF-κB regulated GFP reporter in pancreatic cancer cells. Number of GFP-positive MiaPaCa-2 and PANC-1 cells per field of view after transduction with lentiviral κB-GFP reporter and treatment with either CBL0137 (2μM), or TNF (10ng/ml), or their combinations for 24 hrs. Mean of 10 fields of veiw +/− SDV. B. CBL0137 inhibits expression of NF-κB target genes induced with gemcitabine. qPCR analysis of reverse transcribed total RNA from MiaPaCa-2 cells treated with CBL0137 (2μM) or gemcitabine (GEM, 20μM) for 24 hrs. Mean of three replicates +/− SDV. C-D. CBL0137 inhibits expression of RRM1 and RRM2 subunits of RNR induced by gemcitabine. (C) qPCR analysis of reverse transcribed total RNA from MiaPaCa-2 cells treated with CBL0137 (2μM) or gemcitabine (GEM, 20μM) for 4 or 24 hrs. Mean of three replicates +/− SDV. (D). Western blotting of the lysates of MiaPaCa-2 cells treated as in C for 24 hrs. SSRP1 and SPT16 subunits were probed as a control of CBL0137 effect on FACT.

CBL0137 inhibits FACT function through depletion of the pool of active FACT involved in transcription elongation ([4] and Fig.5D). This leads to the inhibition of transcription regulated by several transcriptional factors whose function depends on FACT, e.g. NF-κB [4]. Activation of NF-κB in tumor cells has been shown to be responsible for resistance to different types of chemotherapeutic drugs, including gemcitabine [15-18]. As demonstrated with several pancreatic cancer cell lines, CBL0137 inhibits NF-κB reporter activity induced by TNF and blocks expression of the endogenous NF-κB targets, IL-8 and TNF, including that which is induced by gemcitabine treatment (Fig.5A, B).

To further investigate the role of CBL0137 in the enhancement of gemcitabine activity, the effect of CBL0137 on the expression of other factors associated with resistance/sensitivity to gemcitabine. These factors belong to several functional classes, including nucleoside transporters (e.g. hENTs), enzymes of nucleotide metabolism (e.g. deoxycytidine kinase (dCK), cytidine deaminase (CDA), ribonucleotide reductase (RNR) subunits RRM1 and RRM2, and general anti-apoptotic factors (e.g. XIAP) (reviewed in [19]). Treatment of human pancreatic cancer cells with CBL0137 resulted in a dose dependent reduction of protein and mRNA levels of RRM1 and RRM2, (Fig.S3A). Importantly, CBL0137 was able to prevent gemcitabine induced expression of RRM1 and RRM2 on mRNA and protein levels (Fig.5C, D). No significant changes across cell lines tested were observed in the expression of ENT1, CDA and dCK protein levels. These data suggest that CBL0137 affects different aspects of gemcitabine resistance most probably through the inhibition of transcription of genes that are induced by gemcitabine treatment and may play a role in gemcitabine resistance.

### CBL0137 is toxic for gemcitabine resistant cancer stem cells

**Figure 6:**
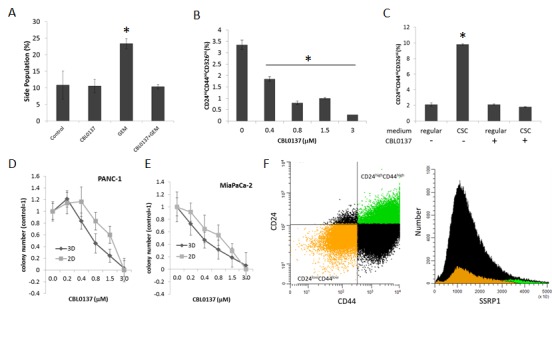
CBL0137 is toxic for cancer stem cells (CSC) A. CBL0137 prevents enrichment of “side population” induced with gemcitabine treatment. Flow cytometry of PANC-1 cells stained with Hoechst 33342 after treatment first with CBL0137 (3μM) for 1 h and then with gemcitabine (20μM) for 72 hrs. B. Reduction of proportion of cells expressing surface markers of CSC in population of PANC-1 cells treated with CBL0137 for 1 h and then left untreated for 72 hrs. C. Assessment of the proportion of cells with CSC surface markers in the population of PANC-1 cells treated or untreated for 1 h with 3μM of CBL0137 and incubated then in regular or CSC media (see Materials and Methods for details). D-E. CBL0137 kills cells able to form colonies in 2D and 3D conditions to a similar extent. Cells were treated in suspension with different concentrations of CBL0137 for 1 h and then plated without the drug in either serum-free semisolid medium for 3D growth or in regular medium on plastic for 2D growth (see Material and Methods). Colonies were counted using inverted microscope. Mean of three replicates +/− SDV. F. The highest level of SSRP1 expression is observed in population of PANC-1 cells positive for markers of CSC (CD24^Hi^CD44^Hi^). Flow cytometry analysis of cells stained with antibodies to CD24, CD44 and SSRP1. Dot plot shows gating of CD24^Hi^CD44^Hi^ cells (green) and CD24^Low^CD44^Low^ cells (orange) which can be seen on histogram of SSRP1 expression in the same cell population. Asterisk shows conditions different from control with p<0.05 (t-test).

It was proposed that the relapse of pancreatic and other cancers after treatment with cytostatic drugs, including gemcitabine, in the clinic may be due to the inability of these drugs to eliminate slowly dividing cancer stem cells (CSC) since their mechanism of activity is dependent on DNA replication [20]. Since the toxic effect of CBL0137 does not depend on cell proliferation, the effect of CBL0137 and gemcitabine on the cancer stem cell (CSC) population was compared. It was shown that treatment of PANC-1 cells with gemcitabine led to enrichment of CSC population, defined as “side population” using flow cytometry because of their ability to efflux Hoechst 33342 dye due to high expression of multi-drug transporters. [21]. Consistent with the literature, gemcitabine caused an increase in the “side population” (Fig. 6A). In contrast, treatment of cells with CBL0137 did not increase the “side population”. Furthermore, CBL0137 prevented the increase of “side population” induced by gemcitabine, suggesting that CBL0137 is as toxic for CSCs as for proliferating tumor cells and, moreover, is able to prevent gemcitabine-induced enrichment of CSCs.

Pancreatic CSCs can also be identified using several surface markers, among which CD24, CD44 and CD326 are the best established [22, 23]. Therefore, to confirm the toxic effect of CBL0137 on CSCs, these cell surface markers were evaluated in PANC-1 cells following treatment with CBL0137. In the experiment, tumor cells were treated with different concentrations of CBL0137 for 1h, and then left to grow for an additional 72h. The number of CSCs was determined by flow cytometry following staining for CD24/CD44/CD326 surface markers. In comparison to control cells, there was a dose-dependent decrease of the CD24^Hi^CD44^Hi^CD326^Hi^ population in cells treated with CBL0137 (Fig. 6B), suggesting that CBL0137 may be even more toxic to CSCs than to the bulk tumor cell population.

Additionally, the ability of CBL0137 to abrogate the accumulation of CSC *in vitro* was investigated using pancreatic tumor cells propagated under conditions specifically developed for CSC enrichment by Benayon and Shaked ([24], see details in Material and Methods). Specifically, the amount of CD24^Hi^CD44^Hi^CD326^Hi^ cells in the population of PANC-1 cells maintained in regular and CSC-enrichment conditions was determined with or without pre-treatment with CBL0137. While the amount of CD24^Hi^CD44^Hi^CD326^Hi^ cells was significantly increased upon propagation in “CSC enrichment” conditions, pretreatment of cells with CBL0137 blocked this increase in CD24^Hi^CD44^Hi^CD326^Hi^ cells (Fig. 6C).

Finally, since one of the critical properties of CSC is ability for anchorage independent growth, we compare effect of CBL0137 on the growth of colonies in 3D (serum free soft agar based medium) and in 2D (on plastic in regular medium). PANC-1 and MiaPaCa-2 cells were treated in suspension with different concentrations of CBL0137 for 1 h and then plated either in 3D or 2D conditions. CBL0137 treatment led to similar dose dependence loss of colony forming ability in both conditions and complete disappearance of cells at 3μM either grown on plastic or in semisolid medium (Fig. 6D, E).

Based on this collection of data, CBL0137 appeared to be toxic for CSCs to the same or even greater extent than to bulk tumor cell population. Since toxicity of CBL0137 cells depends on FACT expression and previous analysis of FACT expression on mRNA and protein levels suggested that it is a marker of normal stem cells [12], FACT expression levels in pancreatic CSCs were determined in order to understand the toxicity of CBL0137 to CSCs. SSRP1 levels were compared between CD24^Hi^/CD44^Hi^ and CD24^Lo^/CD44^Lo^ PANC-1 cell populations. Although SSRP1 is expressed in most PANC-1 cells, its level in CD24^Hi^/CD44^Hi^ cells was significantly higher than in CD24^Lo^/CD44^Lo^, suggesting that indeed FACT is highly expressed in CSCs thereby making these cells sensitive to CBL0137 toxicity (Fig.6F).

## DISCUSSION

CBL0137 is a first in class anti-cancer drug candidate that is an inhibitor of the histone chaperone, FACT [4]. FACT was recently shown to be a novel target in cancer due to its frequent overexpression in different types of cancer and dependence of survival of tumor, but not normal cells, on FACT function [13]. One of the highest rate of FACT overexpression is in PDA, 59% of all tumor samples [13]. However the important question is whether tumors that are negative for FACT (41% of PDA) would respond to CBL0137 treatment. In other words, can FACT be used as a predictive marker of CBL0137 efficacy in clinic?

Establishment of a predictive biomarker of CBL0137 efficacy will allow selection of patient with the highest chance of response while conserving resources of other patients for different types of treatment. Direct expression of the drug target and confirmed dependence of tumor cells on this target are the most reliable predictors of drug activity. In the case of curaxins (i.e. CBL0137), this situation “is complicated”, since although they inhibit FACT, it is not FACT but DNA that is their direct target and DNA is present in all cancer cells. However, since curaxins do not cause DNA damage and do not appear to inhibit DNA-related processes in cell-free systems (i.e., replication, transcription, TF binding to consensus elements ([4] and unpublished data), it is not yet certain that the act of curaxin binding to DNA in itself has any direct anti-cancer effect. Thus, FACT, although an indirect target, is likely the most sensitive factor to curaxins in cells. However, FACT-independent toxicity of curaxins cannot be excluded without testing curaxins in FACT-null cells. Although attempts to model this situation *in vitro* using syngeneic tumor cell pairs demonstrated that toxicity of curaxins to tumor cells was dependent on FACT expression [4], it did not validate FACT as predictive marker of curaxins efficacy. First, artificial modulation of FACT levels in cells did not model natural dependence of tumor cell survival on FACT expression. Second, inhibition of FACT expression in all tumor cell lines tested so far *in vitro* was toxic for these cells [4] and, therefore, FACT-null tumor cells could not be established to test the effect of curaxins on a FACT-null background. Moreover no naturally occurring FACT-null cells were identified among >50 cell lines tested *in vitro*. At the same time, based on the analysis of TMA, it was expected that at least half of the patient pancreatic tumors should be FACT negative [13] and this would therefore make it possible to test CBL0137 efficacy against PDA of different FACT status in mice using PDX models. Contrary to this expectation, all ten PDX samples that were tested were FACT positive (Table 1). This did not appear to be a reflection of the fact that they were passaged in mice since the levels of FACT staining of primary and passaged tumor were similar. It is possible that the surgical samples used for PDX studies contained more heterogeneity due to the size of the sample obtained than the limited available tumor from biopsies that were used on TMAs. These potential explanations require further investigation.

Interestingly, although no FACT negative PDX samples were identified, different degrees of SSRP1 and SPT16 positivity allowed for the observations of some correlations. First, previous tumor analysis used only SSRP1 staining [13], while in the current study, an optimized SPT16 staining procedure was employed to get data for the expression of both FACT subunits. In this study, there was a significant correlation between SSRP1 and SPT16 expression; however, it was lower than for normal tissues analyzed before (r=0.6 vs r>0.9 in normal tissues [12]. This suggests a potential tumor-specific independent function of either of the subunits, which was not evident in normal tissues. Second, consistent with previous data, there was no significant correlation between Ki67 and FACT expression (Table 1), confirming that FACT is not a marker of cell proliferation [12]. Curiously, although the period of time needed for engraftment of PDX samples had a tendency for a negative correlation with the number of proliferating (Ki67+) cells in a sample, this correlation was positive with FACT expression. Therefore, it may be hypothesized that if FACT is a marker of slowly dividing CSC, then samples rich in these types of cells need more time to reach palpable size due to slower proliferation.

At the same time, the limited difference in FACT expression levels between different samples did not allow us to draw accurate conclusions about the correlation of FACT level with sensitivity of PDX samples to CBL0137. The only suggestive observation is that the sample with the lowest level of FACT expression was almost resistant to CBL0137, while the sample with the highest expression of FACT was the most sensitive (partial tumor regression in response to CBL0137 (Table 2). Although this is opposite to what was observed with syngeneic cell pairs with artificially reduced or elevated FACT levels [4], this can be explained from the standpoint of the theory of cancer cell addiction to the activity of certain factor. If the concept of cell addiction to a certain factor is excluded, then elevation of this factor will make cells more resistant to the drug inhibiting this factor and vice versa, which is the situation observed with syngeneic cell pairs. However, in naturally occurring tumor cells, high level of expression may indicate high dependence of cells on the activity of this factor and, therefore, even minimal reduction of its level may significantly affect viability of cells whereas low expression may suggest low or no dependence and, therefore, inhibition of this factor with a drug may have no significant effect on cell viability.

One important aspect of this study is the demonstration that CBL0137 is toxic to pancreatic cancer cells independently of their sensitivity to the standard of care drug gemcitabine. This finding was expected based on the differences in the mechanisms of action of the two drugs. Gemcitabine is a nucleoside analog in which the hydrogen atoms on the 2′ carbon of deoxycytidine are replaced by fluorine atoms. Triphosphate analogue of gemcitabine replaces cytidine during DNA synthesis, which inhibits DNA replication. Additionally the diphosphate analogue of gemcitabine binds to ribonucleotide reductase (RNR) active site and inactivates the enzyme irreversibly. Once RNR is inhibited, the cell cannot produce the deoxyribonucleotides required for DNA replication and repair, and cell apoptosis is induced [25]. Although the induction of apoptosis with gemcitabine treatment in pancreatic cancer cell lines was reported in the literature [26-28], there were no observed biochemical signs of apoptosis in the cell lines used in the current study upon treatment with gemcitabine (Fig.1C, D). Moreover, growth curves of cells in the presence of gemcitabine suggested growth inhibitory effects with no reduction of cell number with increasing gemcitabine dose after a certain concentration and multiple live cells were detected 72 h after the start of treatment even at the highest dose of gemcitabine used (Fig.1A, B). In contrast, CBL0137 treatment cause a clear dose dependent reduction of cells up to complete absence of cells at concentrations >2.5μM, which was confirmed by the presence of all biochemical markers of apoptosis tested (Fig.1A-D).

It was previously determined that CBL0137 does not require cell proliferation for the anti-tumor effect (unpublished data). This was confirmed when CBL0137 was combined with gemcitabine. Although the latter cause strong growth arrest, CBL0137 was still able to induce apoptosis in cancer cells when it was used on the background of gemcitabine treatment (Fig.1C, D). In line with this, in all cases *in vitro* and *in vivo* presented here, improved efficacy of gemcitabine was observed in the presence of CBL0137. This did not depend on whether the pancreatic tumor was resistant or sensitive to CBL0137 alone or gemcitabine alone, suggesting that the combination of two drugs may be useful in many cases when each of them alone does not have potent anti-tumor effect. The reasons for the synergistic activity of two drugs against tumors, but not normal tissues (no significant increase in toxicity was observed even though both drugs were used at close to MTD doses) may be in tumor specific mechanisms of pancreatic cancer cells resistance to gemcitabine, which can be alleviated by CBL0137 treatment. For example, it was reported that constitutive or induced NF-κB activity in cancer cells makes them much more resistant to gemcitabine due to NF-κB controlled expression of several anti-apoptotic factors, such as Bcl2 or IAP family of proteins [16]. In fact, in this study, gemcitabine was a potent inducer of NF-κB transcriptional activity in pancreatic cancer cells and this induction was completely blocked by CBL0137 (Fig. 5A, B).

Surprisingly, this expected mechanism was not the only one observed in PDA cells that might explain the synergistic toxicity of gemcitabine and CBL0137. CBL0137 was also a potent inhibitor of basal and gemcitabine induced expression of RNR subunits RRM1 and RRM2. In addition to blocking DNA repair, inhibition of RNR, the rate-limiting enzyme in deoxyribonucleoside triphosphate (dNTP) synthesis, reduces the endogenous dNTP pool, and indirectly facilitates gemcitabine metabolite incorporation into DNA [29]. The transcriptional upregulation of RRM1 and RRM2 has been consistently observed in pancreatic tumors resistant to gemcitabine [30-32] and pancreatic cancer cell lines [31]. Clinically, low RRM2 mRNA expression levels correlated with significantly enhanced disease-free, median, and overall survival as well as overall response rate in gemcitabine treated patients [33, 34]. The mechanism of how CBL0137 inhibits expression of RRMs is not clear, since little is known about how gene expression of these subunits is regulated.

Finally, similar to the clinical situation, gemcitabine treatment of PDX as a single agent resulted in tumor regression and complete disappearance of tumors in some mice, which was followed by tumor relapse, suggesting that some resistant cells are able to survive gemcitabine treatment and become a source of recurrent tumor. It is believed that CSCs play a crucial role in establishment of drug resistance and relapse of tumors [35, 36]. For many standard of care chemotherapeutic drugs it has been shown that while they eliminate the bulk population of proliferating tumor cells, they stimulate the growth and expansion of CSCs [35, 36]. CBL0137 treatment significantly reduced relapse of PDX after gemcitabine treatment (Fig. 4 and S2), suggesting that CBL0137 may effectively eliminate CSCs. Previously, the indirect target of CBL0137, FACT, was found to be a marker of normal stem cells [12]. The study described here suggests that FACT might also be a marker of CSCs (Fig. 6D). If FACT is a specific target of CSCs, then drugs targeting FACT, such as CBL0137, become promising candidates for CSC elimination. Indeed, CBL0137 was able to inhibit gemcitabine-induced CSC enrichment, which, together with the *in vivo* data, makes CBL0137 a reasonable and promising adjuvant for gemcitabine: while the latter eliminates bulk population, the former kills CSCs thereby preventing reoccurrence of cancer.

## MATERIALS AND METHODS

### Drugs and reagents

Dimethyl sulfoxide was purchased from Fisher Scientific and Captisol^®^ was obtained from CYDEX Pharmaceuticals, INC. (Lenexa, KS). CBL0137 was provided by Incuron, LLC (Buffalo, NY) as 20mM solution in DMSO for *in vitro* experiments or 6.25-11.25 mg/ml solution in 100 mg/ml captisol for *in vivo*. Gemcitabine was purchased from LC Laboratories (Woburn, MA). Gemcitabine was dissolved in DMSO at 20mM for *in vitro* studies, and in sterile water at 2 or 4 mg/ml for *in vivo* studies. FAM-labeled real-time PCR primer/probe sets for RRM1, RRM2, IL-8, TNF and β-microglobulin as well as Taqman universal master mix were purchased from Life Technologies (Grand Island, NY). Matrigel was from Corning (Corning, NY).

Antibodies against the RRM1 and RRM2 subunits of ribonucleotide reductase and all secondary antibodies were obtained from Santa Cruz Biotechnology (Dallas, TX). Antibodies to caspases and PARP-1 were from Cell Signaling Technology (Danvers, MA). SSRP1 and SPT16 antibodies were from Biolegend (San Diego, CA).

The following antibodies were used for flow cytometry: PE Mouse Anti-Human CD24 and Isotype control PE Mouse Anti-Human IgG clone G18-145 (BD Pharmingen, Piscataway, NJ); PE-Cy7 Mouse Anti-Human CD44, clone G44-26 and PE-Cy7 Mouse Anti-Human IgG Clone G18-145 (BD Pharmingen, Piscataway, NJ); CD326 (EpCAM)-FITC, human and Isotype control Anti-IgG-FITC, human (MACS Miltenyi Biotec, San Diego, CA); SSRP1 (# ab21584, Abcam, Cambridge, MA); Alexa Fluor 488 donkey anti-rabbit IgG (H+L), (Invitrogen, Burlington, ON).

### Cell lines and cell culture

MiaPaCa-2, BxPC-3, PANC-1 and H1975 human pancreatic cancer cell lines were purchased from ATCC. MiaPaCa-2 and PANC-1 were maintained in Dulbecco's Modified Eagle Medium (DMEM) supplemented with 10% fetal bovine serum (FBS). BxPC-3 and the H1975 non-small cell lung cancer cell line were maintained in RPMI 1640 medium containing 10% FBS.

### Cytotoxicity assay

Cells were plated in 96 well plates at 10-20% confluency. After overnight incubation, drugs were added to cells as ten 2-fold serial dilutions. Control for no toxicity was 0.1% DMSO and for complete cell death - 50μM solution of 9-aminoacridine. All treatments were done in triplicate. Cell viability was assessed at 72 hrs after start of treatment using Cell Titer Blue Assay (Promega, Madison, WI). Mean readings from wells with 9-aminacridine were subtracted from all other wells, after that cell viability was calculated at mean reading of three replicates for all treated conditions relative to mean reading of wells treated with 0.1% DMSO.

### Colony assay

Cells were plated at 10^3^ per well of 6-well plate in triplicates. After attachment, cells were treated with drugs for 4 hrs and then medium was changed to drug-free medium. The number of colonies was counted 10-14 days later using methylene blue staining.

### Comparison of cell growth in 2D and 3D conditions

Cells were resuspended in serum free DMEM and treated with different concentrations of CBL0137 for 1h. After that 10^5^ from each treatment condition were plated in 3 wells of 6-well plate in 2ml of serum-free DMEM/F12 medium (Gibco, Grand Island, NY) supplemented with 0.4% BSA, 0.2 × B27 (Invitrogen, Grand Island, NY), 10 ng/ml recombinant EGF (Sigma-Aldrich) and containing 0.25% agarose. 10^3^ cells from each treatment condition were plated in 3 wells of 6-well plate in regular FBS containing medium. Colonies were counted using inverted microscope 7-15 days after plating.

### Propagation of cells for CSC enrichment

This experiment was performed according to [24]. In brief, 70% confluent PANC-1 cells were pre-treated for 1h with 1μM CBL0137 or left untreated. When cells reached 100% monolayer, they were re-plated in serum-free DMEM:F12 medium (Gibco, Grand Island, NY) supplemented with Growth Factor (GF) cocktail (80μl of 0.4%BSA; 1.85μl of 20ng/ml EGF; 1.85μl of 10 ng/ml bFGF; 1.85μl of 5 μg/ml insulin; 0.05 μl of ITS supplement for 1ml of DMEM:F12 medium) and let grow for 2 days. Then floating cell spheres enriched for CSCs were collected and transferred to a new plate supplemented with fresh DMEM:F12/GF medium, the initial plate was also supplemented with fresh DMEM:F12/GF medium. Sphere collection procedure was repeated 2 more times with a 2 day interval after which all cells were collected for FACS analysis.

### Stem cell identification using the Hoechst 33342 side population (SP) assay

Assay was done according to [37-39] with several modifications. PANC-1 cells were collected by trypsinization, and then trypsin was inhibited by FBS. Cells were then washed with 1xPBS and re-suspended in 1xHanks Buffer supplemented with 1% of FBS. Verapamil (100μM, (Krackler Scientific, Albany, NY)) or KO-143 (1μM, kindly provided by Dr. W. Huss, Roswell Park Cancer Institute) were used for inhibition of drug transporters in “no-SP”- control samples: 15 minute, 37°C. Control and experimental cell samples were incubated with Hoechst 33342 (5μg/ml) for 1.5h at 37°C with gentle pipetting every 15 minutes. Cells were than pelleted and the pellet was resuspended in 1x PBS and analyzed by FACS. FACS analysis was performed at RPCI flow cytometry facility on a LSA II UV A flow cytometer (BD Biosciences, San Jose, CA). For each cell line, the proper scatter parameters were identified and then cytometry was performed accordingly using BlueA 645 LP filter with a 502LP mirror. At least 100,000 events (singlets) were collected for each sample. The data were analyzed using the WinList 3D program (Verity Software House, USA). Side population location was estimated for each treatment condition using Verapamil/Ko143-pretreated samples.

### Stem cell identification using surface markers

Cells were collected by trypsinizing and resuspended in ice-cold Phospahte Azide Buffer (PAB) consisting of 0.1% Sodium Azide, 2% BSA in 1xPBS. Human TruStain FcX reagent (BioLegend, San Diego, CA) diluted 1:10 was used to inhibit unspecific staining (10min incubation on ice), cells were than stained with combinations of antibodies to cell surface markers or their isotype control antibodies diluted with PAB (30min-1h staining on ice in the dark).

For SSRP1 staining samples were fixed and permeabilized after staining with antibodies to cell-surface markers using Fixation/Permeabilization System (eBioscience, San Diego, CA). After that SSRP1 staining was performed with specific primary and secondary antibodies.

FACS analysis was performed on LSR Fortessa A cytometer (BD Biosciences, San Jose, CA). For each sample at least 100000 events were collected. Obtained data was analyzed by WinList 3D program (Verity Software House, USA).

### Gene expression analysis by real-time PCR

MiaPaca2 and BxPC-3 cells were treated with CBL0137 alone or in combination with gemcitabine for 4 or 24h. 0.1% DMSO served as vehicle control. After the incubation period, cells were harvested and RNA isolated using the RNeasy Mini Kit (Qiagen). cDNA was prepared using 2 μg RNA, Superscript II reverse transcriptase (Life technologies, Grand island, NY) and random hexamers [40]. Expression of RRM1. RRM2, IL-8 and TNF were determined using Taqman universal master mix with β_2_-microglobulin used as an internal control. The PCR reaction consisted of an initial incubations of 50°C for 2 minutes and 95°C for 10 minutes followed by 40 cycles of 95 °C for 15 seconds and 60°C for 1 minute. PCR data were collected with the use the Applied Biosystems 7300 real-time PCR system. The level of target gene expression was determined using the ΔΔCt method where the DMSO control for each comparison was used as the calibrator [41].

### protein expression analyses

MiaPaca2 and BxPC-3 cells were treated with CBL0137, gemcitabine or a combination of the two for 4 or 24h. Cells were harvested in 1x Cell Culture Lysis Reagent (Promega, Madison, WI) containing protease (Roche) and phosphatase inhibitors (Sigma-Aldrich). Lysates 5-20 μg were separated on SDS-PAGE gels and transferred to PVDF membranes. Blots were probed with antibodies specific for SSRP1, SPT16, RRM1, and RRM2. GAPDH was used as a loading control. Proteins were visualized using ECL kit (GE Healthcare).

Immunohistochemical (IHC) and hematoxylin and eosin (H&E) staining was done in the Pathology Resource Core of RPCI as already described [12].

### Animal studies

All animal studies were performed with approval of the IACUC of RPCI and State University of New York Downstate Medical Center. SCID female 5-10 weeks old mice from DLAR RPCI or athymic nude mice from Harlan were used.

**PANC-1 orthotopic model:** 10-week old female athymic nude mice (n=8 per treatment group) were deeply anesthetized with ketamine/xylazine. Using laparotomy, 2 × 10^6^ PANC-1 cells were inoculated into the tail of the pancreas of each mouse. Two weeks following inoculation (tumor presence confirmed by ultrasound), treatment commenced. Mice were treated for 4 weeks. One week after treatment was finished, mice were euthanized, the tumors measured upon necropsy and then pancreas with tumor was fixed and stained with H&E for histological analysis.

**Patient derived xenografts (PDX) model.** Pancreatic tumor surgical samples were obtained with approval from the Institutional Review Board of RPCI. For *in vivo* efficacy studies, the patient derived tumors were passaged through severe combined immunodeficient (SCID) mice (RPCI Laboratory Animal Resource) as already described [4] with some modifications. Frozen tumor pieces (8-10mm3) were thawed on ice and after that transplanted into two flanks of 2 donor SCID mice subcutaneously. 2-3 additional tumor pieces were washed in cold PBS and fixed in 10% buffered formalin for H&E and IHC staining. When any of tumors in donor mice reached 500mm3 the tumor was excised from the anesthetized mouse, washed with PBS and cut into 8-10mm3 pieces and re-transplanted in 10 recipient mice. Again part (1/3-1/2) of tumor sample was used for H&E and IHC staining. For combination studies, 2-5 mm pieces were implanted into each flank of SCID mice. For all PDX studies, treatment was started when at least one tumor per mouse reached ~50 mm3. Mice were distributed between groups using alternating method. Mice were monitored daily, weighed 3-5 times per week and tumor size was measured with digital caliper a minimum of twice per week.

**H1975 non-small cell lung cancer cells** (5×10^6^,) were inoculated in a single flank of athymic nude mice as a 1:1 mixture with matrigel. Treatment commenced when tumors reached ~150-200 mm^3^. Mice were followed until tumors reached 2000 mm^3^ or 7 weeks from start of treatment.

**Treatments:** The following regimens were used: 1) vehicles, 100mg/kg captisol i.v. and sterile water via gavage, 2) 50- 90 mg/kg CBL0137 in 100 mg/ml captisol i.v. delivered via tail vein once per week, 3) 10-20 mg/kg CBL0137 p.o. via oral gavage, 5 days on/2 days off, 4) 20 or 40 mg/kg gemcitabine in sterile water i.p. every fourth day or 4) CBL0137 and gemcitabine at the indicated regimens. Mice were treated for 4 weeks.

**Tumor measurement** was done with digital calipers. Tumor volume was calculated using the equation L x W^2^/2 where L is the longest dimension and W is the dimension perpendicular to W [42]. Mice were followed until at least one tumor per mouse reached 1000 mm^3^ or 90 days from start of treatment, whichever came first. Mean fold tumor growth was calculated relative Day 1 (tumor volume Day X/tumor volume Day 1). Comparisons of tumor growth across groups were performed using ANOVA (GraphPad Prism 6).

## SUPPLEMENTARY MATERIAL AND FIGURES


