# Supplementary material for: Curaxin CBL0137 eradicates drug resistant cancer stem cells and potentiates efficacy of gemcitabine in preclinical models of pancreatic cancer
Source: Oncotarget. 2014 Nov 6;5(22):11038–53. doi: 10.18632/oncotarget.2701 (PMC4294371; doi:10.18632/oncotarget.2701)
Supplement: Supplementary file 1 [file oncotarget-05-11038-s001.pdf]

Curaxin CBL0137 eradicates drug resistant cancer stem cells and potentiates efficacy of gemcitabine in preclinical models of pancreatic cancer

Supplementary Material

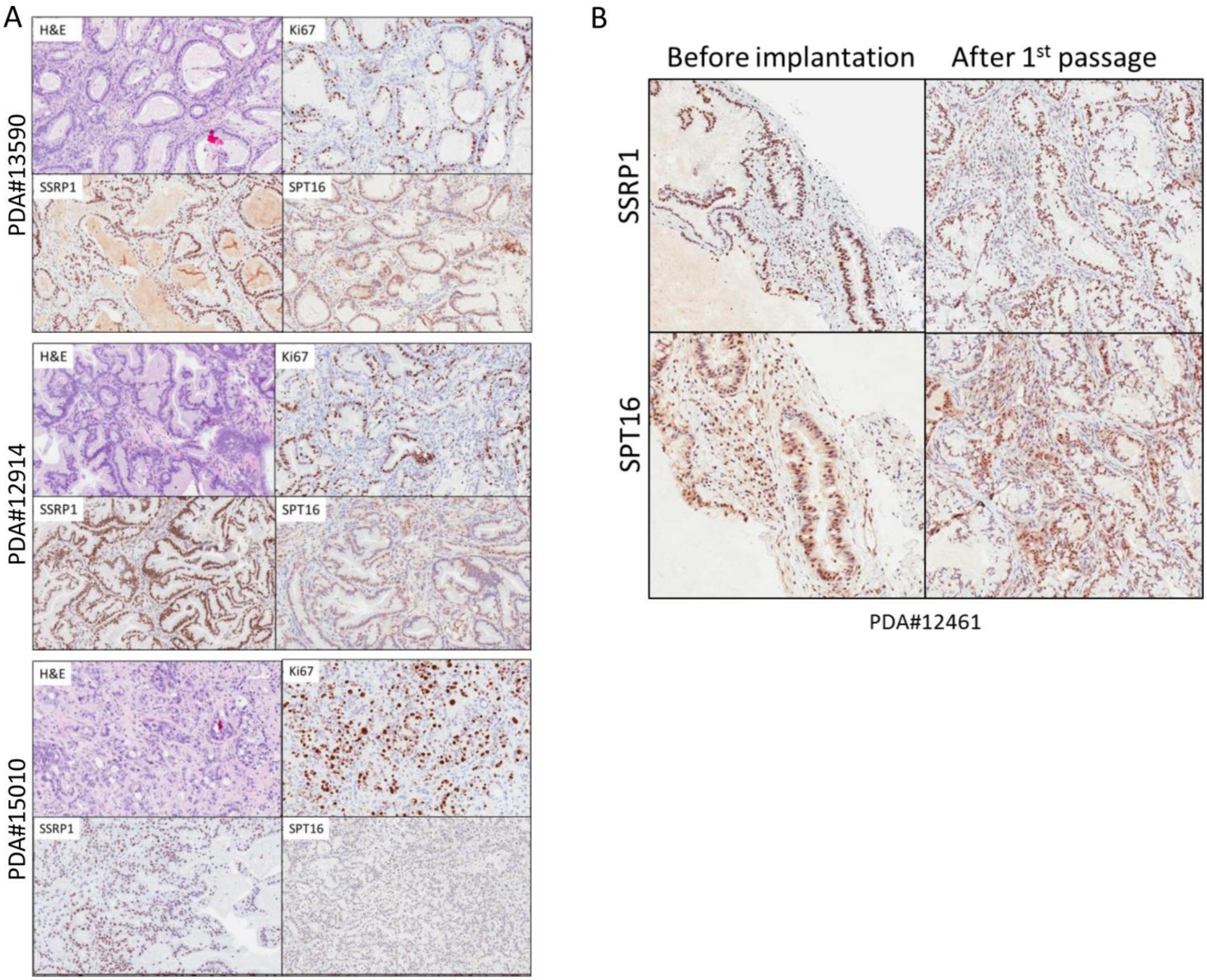

Figure S1. Morphology and expression of FACT subunits (SSRP1, SPT16) and proliferation marker Ki67 in PDX samples of pancreatic ductal adenocarcinoma (PDA) used in the study. H&E and IHC staining with indicated antibodies. A. Additional PDA samples with high to intermediate FACT expression. B. Comparison of morphology and SSRP1, SPT16 expression of the same sample before and after passage in donor mice.

A.

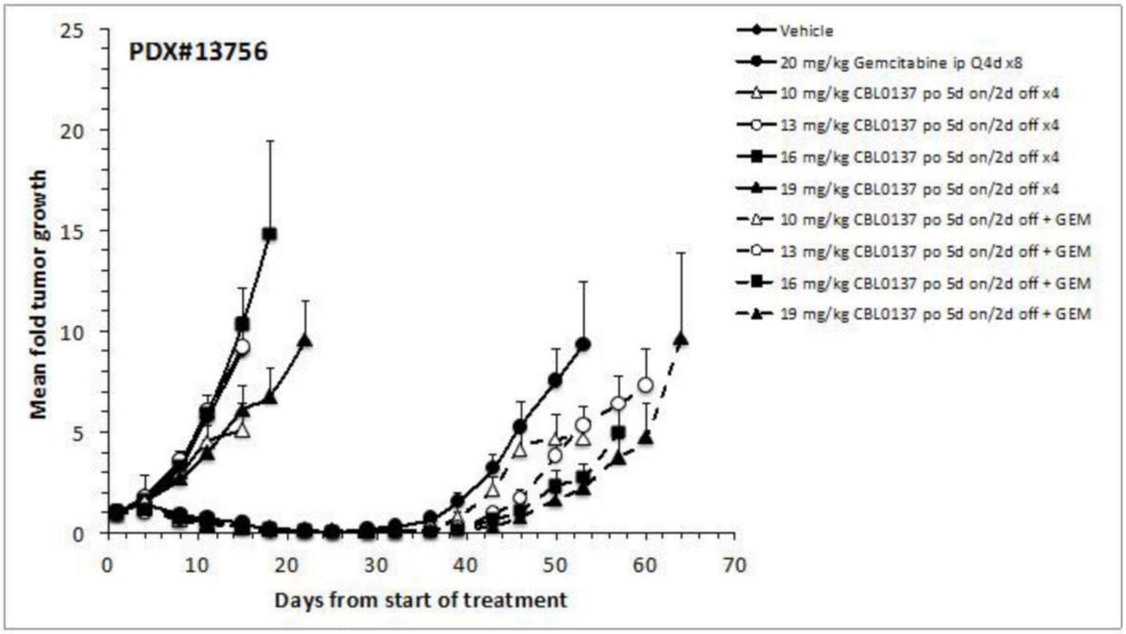
PDX#13756
Mean fold tumor growth
Days from start of treatment
Vehicle
20 mg/kg Gemcitabine ip Q4d x8
10 mg/kg CBL0137 po 5d on/2d off x4
13 mg/kg CBL0137 po 5d on/2d off x4
16 mg/kg CBL0137 po 5d on/2d off x4
19 mg/kg CBL0137 po 5d on/2d off x4
10 mg/kg CBL0137 po 5d on/2d off + GEM
13 mg/kg CBL0137 po 5d on/2d off + GEM
16 mg/kg CBL0137 po 5d on/2d off + GEM
19 mg/kg CBL0137 po 5d on/2d off + GEM

B.

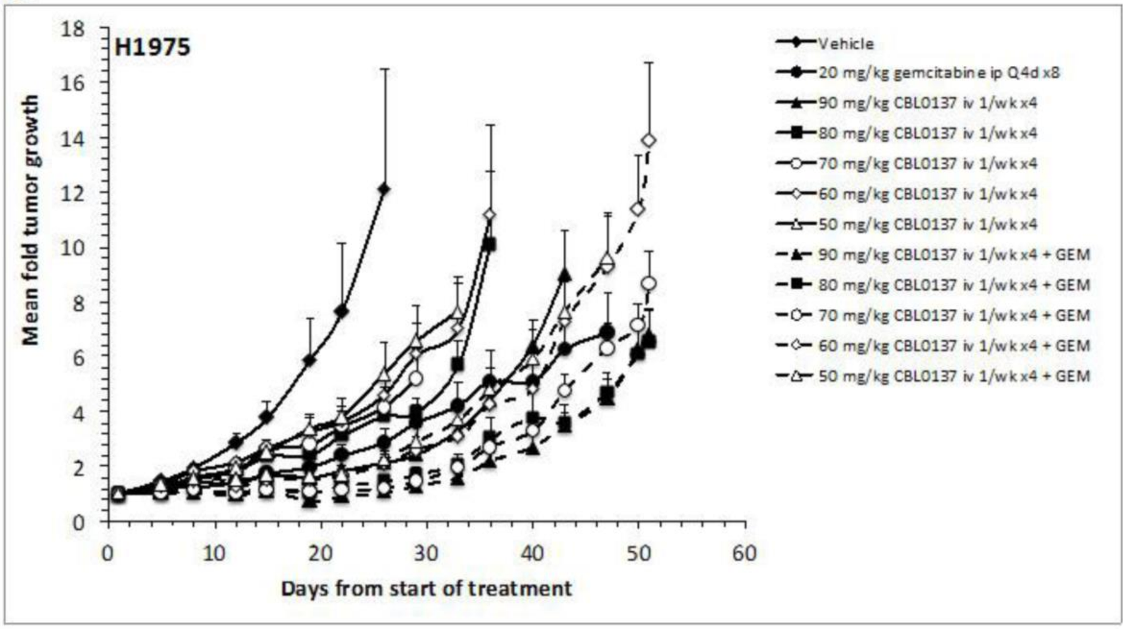
H1975
Mean fold tumor growth
Days from start of treatment
Vehicle
20 mg/kg gemcitabine ip Q4d x8
90 mg/kg CBL0137 iv 1/wk x4
80 mg/kg CBL0137 iv 1/wk x4
70 mg/kg CBL0137 iv 1/wk x4
60 mg/kg CBL0137 iv 1/wk x4
50 mg/kg CBL0137 iv 1/wk x4
90 mg/kg CBL0137 iv 1/wk x4 + GEM
80 mg/kg CBL0137 iv 1/wk x4 + GEM
70 mg/kg CBL0137 iv 1/wk x4 + GEM
60 mg/kg CBL0137 iv 1/wk x4 + GEM
50 mg/kg CBL0137 iv 1/wk x4 + GEM

Figure S2: Dose-dependence of CBL0137 enhancement of gemcitabine efficacy. (A) PDX#13756 was inoculated into each flank of SCID mice (n=10/group). When at least one tumor per mouse reached ~50 mm3, treatment began with vehicle, 10-19 mg/kg CBL0137 po 5 days on/2days off, 20 mg/kg gemcitabine ip Q4d or combination of CBL0137 and gemcitabine. Mice were treated for 4 weeks. Mice were followed for up to 90 days from start of treatment or when at least one tumor per mouse reached 1000 mm3. Mean fold tumor growth was calculated by normalizing the tumor volume on Day X to that on Day 1 for each individual tumor and then averaging the normalized values for all tumors in each group at the designated time points. Error bars represent the standard error of the means. (B) Athymic nude mice (n=10) were inoculated in one flank with 5x106 H1975 cells mixed 1:1 with Matrigel. When tumors reached 150-200 mm3, treatment commenced with 90 mg/kg CBL0137 iv 1/week, 20 mg/kg gemcitabine ip Q4d or combination of CBL0137 and gemcitabine. Mice were followed for 8 weeks from start of treatment or when tumors reached 2000 mm3, whichever came first. Data is presented as described in A.

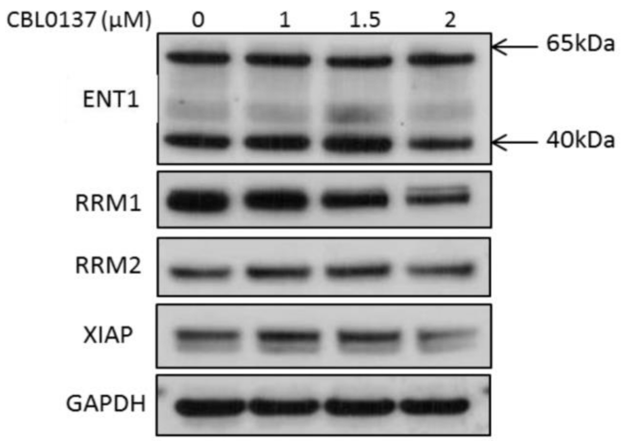

Figure S3. CBL0137 treatments reduces levels of several protein associated with resistance to gemcitabine. Western blotting of lysates of MiaPaCa-2 cells treated with different concentrations of CBL0137 for 24 hrs.
